# Design and Implementation of a Hybrid Optical Camera Communication System for Indoor Applications

**DOI:** 10.3390/s24010300

**Published:** 2024-01-04

**Authors:** Huy Nguyen, Nam Tuan Le, Duy Tuan Anh Le, Yeong Min Jang

**Affiliations:** 1 Department of Electronics Engineering, Kookmin University, Seoul 02707, Republic of Korea; ngochuy@kookmin.ac.kr (H.N.); tuananhduy@kookmin.ac.kr (D.T.A.L.); 2 Advanced Remanufacturing and Technology Centre, Agency for Science Technology and Research, Singapore 637143, Singapore; le_nam_tuan@artc.a-star.edu.sg

**Keywords:** optical camera communication (OCC), hybrid OCC waveform, IoT application

## Abstract

Optical wireless communication is a promising emerging technology that addresses the limitations of radio-frequency-based wireless technologies. This study presents a new hybrid modulation method for optical camera communication (OCC), which integrates two waveforms transmitted from a single transmitter light-emitting diode (LED) and receives data through two rolling shutter camera devices on the receiver side. Then, a smart camera with a high-resolution image sensor captures the high-frequency signal, and a low-resolution image sensor from a smartphone camera captures the low-frequency signal. Based on this hybrid scheme, two data streams are transmitted from a single LED, which reduces the cost of the indoor OCC device compared with transmitting two signals from two different LEDs. In the proposed scheme, rolling-shutter orthogonal frequency-division multiplexing is used for the high-frequency signals, and M-ary frequency-shift keying is used for the low-frequency signals in the time domain. This proposed scheme is compatible with smartphone and USB cameras. By controlling the OCC parameters, the hybrid scheme can be implemented with high performance for a communication distance of 10 m.

## 1. Introduction

Owing to the rigorous requirements for high-rate communications, technological improvements continue to increase in efficiency and overall performance. Communication systems based on wireless technologies are superior to wired communication systems because they are easier to construct and allow for data transmission without wires. However, wireless technologies using radio frequencies (RFs) are used so frequently that they have saturated the frequency resources. Therefore, a higher frequency band is being used to increase the data rates. Many researchers are studying fifth generation (5G) mobile networks in the millimeter-wave frequency band, achieving data rates of 1–10 Gbps. However, higher frequencies cause harmful effects to human health [1].

Lately, there have been investigations into utilizing visible light waves for data transmission featuring three novel candidates: visible light communication (VLC), light fidelity (LiFi), and optical camera communication (OCC). These technologies can be used as substitutes for RF communications. The advantages of visible light waveforms over RF for data transmission are as follows:

- Visible light waves do not harm human health if appropriate dimming and non-flicker methods are used. As mentioned in some results based on human health [2], if the optical modulation frequency exceeds 200 Hz, there is no adverse impact on human eyes.
- The visible light bandwidth is 1000 times larger than the RF bandwidth.
- VLC is more cost-efficient than RF communication; because visible light already exists in the light infrastructure of streets and vehicles, the implementation cost is lower [3].

Optical wireless communication (OWC) technologies are standardized in the Institute of Electrical and Electronics Engineers (IEEE) 802.15.7-2011 standard [4] and IEEE 802.15.7-2018 standard [5] for the aforementioned candidates, namely VLC, LiFi, and OCC. Unlike VLC and LiFi, which use photodiodes, OCC technology uses cameras to receive data. Previous studies [6,7] have determined that OCC system performances depend on the camera type and image quality. Two widely used camera types are presently available, namely rolling-shutter and global-shutter cameras [8,9]. In the case of the global-shutter camera, the OCC data rate is contingent on the camera’s frame rate, ensuring that the sampling rate meets the Nyquist sampling criterion. On the other hand, in the rolling-shutter camera, the sampling rate is influenced by both the camera frame rate and rolling rate. VLC and LiFi have higher data rates owing to the use of photodiodes, whereas OCC has a lower data rate owing to the use of image sensors to receive the data [10,11]. Nevertheless, the OCC system is preferable for applications requiring environmental mobility and long-range usage. The OCC system can be used for vehicle-to-vehicle (V2V) applications, indoor localization, and internet of things applications. In [12], drone-to-vehicle communication was proposed using rolling shutter camera, which achieved a data rate of 3.6 kbit/s with a free-space transmission distance of 23 m. High-speed optical camera V2V communications also proposed in [13] provide a data rate of up to 3.456 kbps by using RaspiCam. An OCC indoor positioning system based on flat panel light and angle sensor assistance was also proposed in [14], which can reach the average positioning error of 6.5023 cm. An optical spatial localization algorithm achieved an error of 0.1 m at 3 m distance in [15]. OCC technology for IoT was also proposed in [16] using an 8 × 8 LED matrix, which achieved 2.25 kbps. In [17,18], the authors proposed a hybrid OWC scheme for IoT with a maximum distance of 5 m.

The orthogonal frequency-division multiplexing (OFDM) technique is utilized to digitally encode data across numerous carrier frequencies. OFDM is often employed in high-speed communications owing to its partitioning of bandwidth into orthogonal subcarriers, which minimizes interference-related distortions. Using Fourier transform (FT), OFDM subcarriers can be overlapped without compromising the signal quality. Additionally, a cyclic prefix (CP) is used to mitigate ISI due to the multipath effect. In [19], a screen OCC system was presented based on 2D-OFDM with a data rate up to 50 kbps. However, the 2D-OFDM scheme uses a screen to transmit data, which is too big and heavy for it to be suitable for an IoT system. In [20], a rolling shutter OFDM (RS-OFDM) was proposed that had the advantages of the rolling-shutter effect of a rolling-shutter camera to transmit OFDM waveforms, which can achieve a high data rate. Hence, RS-OFDM is a good candidate for high data-rate streaming in our work. A hybrid VLC/OCC was also proposed in [21], in which a VLC signal was used with a different frequency to transmit a hybrid waveform at the high OCC signal level, which can transmit data at short distances using photodiode. A hybrid OOK and ACO-OFDM approach was presented for the VLC system in [22], in which the negative and positive clipping parts were used to broadcast two separate waveforms. If the signal-to-noise ratio (SNR) is low over a lengthy communication distance, this indicates certain issues with the ACO-OFDM signal in the low-level strength of the OOK signal. In [22], the photodiode can receive and separate two waveforms in a single photodiode with a difficult to decide threshold OOK signal in the long distance due to decreasing SNR values. The present work proposes a hybrid OCC waveform that combines two OCC schemes, namely RS-OFDM and continuous M-ary frequency-shift keying (CM-FSK). The proposed scheme uses two cameras to receive two different waveforms from a single light-emitting diode (LED) and is referred to as a hybrid OCC scheme herein. This hybrid scheme shows good performance compared with the conventional scheme, in which only one signal is transmitted. Hence, the proposed scheme has good performance compared with other hybrid schemes in [21,22] due to achieving a long distance with a low BER value and is compatible with many types of rolling shutter camera on the market (CCTV, webcam, etc.). Currently, reconfigurable intelligent surfaces (RISs) [23] emerge as good candidates to improve the OWC system through the incorporation of meta-surface structures that are programmable and modifiable to generate a precise reaction to incoming signals. Intelligent reflecting surfaces are a good option for LiFi and VLC based on photodiodes. However, OCC is based on received picture, which is collected by the image sensor. The current RISs devices do not support OCC; however, it is good idea to keep in mind for the next generation of OCC in the future. When RISs devices look like smart mirrors, they will be compatible with OCC systems.

The hybrid OCC waveform proposed here transmits two waveforms (a low data-rate stream and a high data-rate stream) from a single LED. Experimental results are presented to confirm that the proposed scheme can achieve high performance. While the hybrid OCC scheme is not a new concept, the main contributions of this study are as follows:

- It contains a proposal for a new hybrid waveform combining two types of OCC waveforms, a low frame-rate stream and a high frame-rate stream, that are transmitted from a single LED. The two signals are transmitted simultaneously through the same optical channel with varying data rates by combining the two waveforms. Therefore, two different pieces of information from the two systems can be easily transmitted at the same time.
- Increased throughput: The total throughput of the proposed hybrid scheme is calculated by summing the individual throughputs from the two waveforms. The throughput is thus improved when compared with those of conventional schemes.
- Support for frame-rate variation: In an OCC system, frame-rate variation can be quite unpredictable. While many assume that a camera’s frame rate is fixed, such as 30 fps or 1000 fps, each camera has a unique frame rate that is determined by its technical parameters. This variability adds complexity to the task of synchronizing the transmission (Tx) and reception (Rx). However, by utilizing the sequence number in the OFDM scheme and employing the Ab bit in the FSK scheme [5], any receiver with a frame rate greater than the transmitter’s packet rate can easily decode data by checking the sequence number value.
- Data merger algorithm: The sequence number and Ab bit support to address frame-rate variation and advance the OCC performance. This idea is to merge the packages into a whole data sequence in the right order.
- By using a single LED to transmit two different data streams, we can reduce cost while providing various services to users through low-complexity light sources in the communication network.
- Detecting missing packets: When the sequence number length in the OFDM scheme exceeds a certain threshold, it becomes simple to detect any missing packets by comparing the sequence numbers of two consecutive images captured by the camera.
- In an OCC system, it can be challenging to deal with complex noise, which includes issues like blurred images, interference, and irregular signal attenuation in the time domain. Nonetheless, these problems can be effectively addressed in the frequency domain by excluding the DC component of the high data-rate stream using the RS-OFDM scheme.
- The complete hybrid OFDM–FSK symbols at the Tx are as follows: new physical protocol data unit format for the hybrid scheme and design of the pilots and channel equalization for RS-OFDM scheme.

The remainder of this manuscript is organized as follows: Section 2 introduces the system architecture of the proposed approach; Section 3 demonstrates the practical results of the hybrid OCC scheme; and Section 4 entails the concluding remarks of this work.

## 2. System Architecture

**Figure 1:**
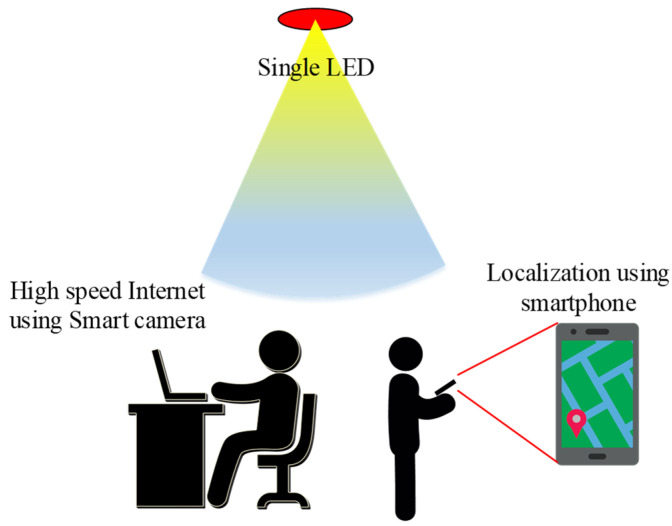
Example of hybrid OCC applications.

An OCC system mainly generates the intensity through which information is encoded in an optical signal. A potentially effective modulation method can improve the system’s communication capabilities. An RS-OFDM scheme is used in the high data-rate stream in the proposed system, while the CM-FSK scheme is applied to the low data-rate stream. This section explains the different characteristics of the two OCC signals and the creation of the hybrid OCC system. The schematic of the hybrid scheme is as shown in Figure 1.

### 2.1. OFDM Scheme

Based on the concept of orthogonality, the FT was proposed in 1966 as a substitute for a sinusoidal bank. The CP was proposed as an addition to the OFDM system in 1969 to combat intersymbol interference (ISI). Researchers began deploying OFDM in wireless communications in 1980. The OFDM is calculated based on the FT as follows:

(1) $$X(\omega )=\sum_{i=-\infty }^{+\infty }x[n]e^{-i\omega n}$$

In an OWC system, especially an OCC system, the signals are encoded according to the intensity of the light sources, and nonnegative values are required. Thus, the OFDM signal should be preprocessed before applying the inverse discrete FT. At present, asymmetrically clipped optical OFDM (ACO-OFDM) and DC-biased optical OFDM (DCO-OFDM) are two popular technologies for VLC/LiFi systems [24,25]. Regardless, compared with the FT, the wavelet OFDM provides many advantages, such as no redundancy in the CP, decreased subchannel interference, and increased spectral separation. The IEEE Standard Association has standardized OFDM technology based on the wavelet transform. The advantage of the wavelet OFDM over another technology has been verified in [26], where it is demonstrated that wavelet OFDM has the spectral benefit that suppresses sidelobes and bit error rate (BER) performance better than the DCO-OFDM. In [27], wavelet OFDM applications of OWC systems are shown, in which the peak-to-average power ratio of the wavelet OFDM is lesser and greater when handling channel conditions.

### 2.2. M-FSK Operation

M-FSK modulation technology for OCC was standardized in IEEE 802.15.7-2018 [5] based on the rolling-shutter effect. FSK is a frequency modulation scheme that transfers information through discrete frequency-signal changes [28]. In [28], the authors proposed M-FSK for a rolling shutter using a smart camera, which can achieve 7 m distance. The M-FSK OCC scheme is a frequency-shift on–off keying modulation that operates with multiple frequency shifts as the light source is turned on and off. The conventional CM-FSK scheme is based on the on/off statuses of the LED, which are represented as the light signal’s intensity at each pixel. The LED transmits modulated data symbols that are represented by different on/off frequencies of the light source. A rolling-shutter camera then receives these with different roll pixel sizes. The bit classification is defined based on the subcarrier waveform frequency (represented by a group of rolling stripes at the image sensor).

**Figure 2:**
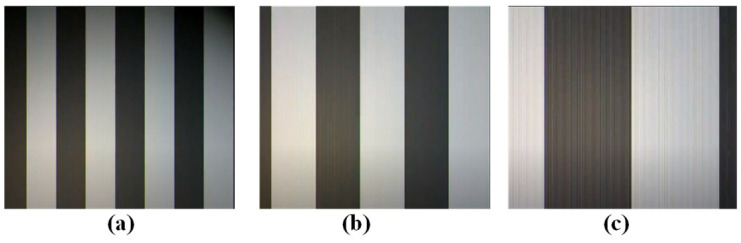
M-FSK signals at (**a**) 2 kHz, (**b**) 1 kHz, and (**c**) 500 Hz.

Different frequencies correspond to various stripe features. However, given a subcarrier frequency, the width of the generating stripe will not be affected by the location, orientation, or size of the light source. To identify the subcarrier frequency of the image sensor’s captured image, fast Fourier transform is used to calculate the frequency of pixel intensity or image processing mechanisms that measure the widths of the stripes. Figure 2 represents the captured image of an M-FSK-modulated signal at different subcarrier frequencies. The quantity of frequency alterations dictates how many bits are embedded in a single symbol. Meanwhile, the number of assigned frequencies specifies the number of bits concealed within a specific optical symbol signal frequency. *F* is the set of allocated frequencies for modulation, so each symbol represents $\log_{2}F$ embedded bits. The system can increase the data rate with more applied frequencies. The rolling-shutter system constrains the number of frequencies in the M-FSK scheme and the number of multiple light sources. The number of pixel rows in the captured image varies inversely with distance for different communication distances. The system can accommodate various types of rolling-shutter image sensors, each with potentially different frame rates, sampling rates, and rolling-exposure durations. In this scheme, two brightness levels of the LED are used, not only for the on and off statuses but also for low and high brightness levels. The purpose of this is to enhance the system data rate. Because of the two brightness statuses, the OFDM signal is embedded in all FSK signal envelopes. This cannot be applied in the OFF status of the LED.

The designed bandwidth and frequency separation of the system should be considered for compatibility with the image sensor, as defined by Equation (2):

(2) $$N=\frac{B}{\Delta f}$$

where *B* is the upper bound of the modulation bandwidth, and Δf is the frequency separation configured based on the camera parameters. The allocated frequencies also affect the maximum communication distance [5], which is represented by Equation (3).

(3) $$d=\frac{\omega \Delta f}{f_{s}}\left(\frac{L}{2\tan\left(\frac{FoV}{2}\right)}\right)$$

where *d* is the communication distance, $f_{s}$ is the rolling rate of the image sensor pixel rows, *L* is the normalized length of the light source, and ω is the rolling-axis image width.

### 2.3. Hybrid OCC Scheme

**Figure 3:**
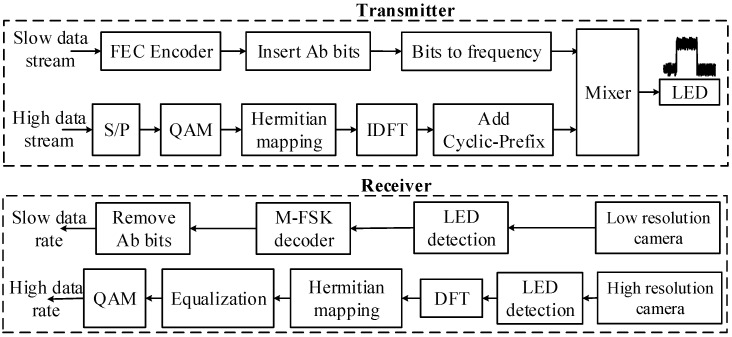
System architecture of the proposed hybrid scheme.

As shown in Figure 3, a hybrid OCC system is proposed herein that can communicate with both high and low data-rate streams using the same light source. Two OCC signals are transmitted at the same time through the hybrid waveform. This proposed system reduces costs while providing various services to users via a low-complexity light source for the communication network. The amount of energy consumed is reduced as the required number of lamps for the communication system are reduced.

In the hybrid OCC scheme, OFDM packets of high-frequency OCC signals are transmitted during the high and low levels of the FSK signal. In [21], the authors proposed a hybrid OCC system that allowed transmission of VLC signals during the high period of the OCC signal. In the proposed scheme, the high-frequency OCC signal is transmitted during both the high and low periods of the FSK signals. Therefore, the data rate increases by two times compared with that of the conventional system. On the receiver side, two cameras detect the low-frequency and high-frequency signals, and this controls the exposure times for both the high-frequency and low-frequency waveforms. The image sensor functions as a lowpass filter, meaning that extending the exposure time results in the attenuation of the high-frequency signals. As the exposure time increases, the communication bandwidth diminishes, leading to a reduction in the overall noise power distributed across the bandwidth.

- *Pilot*

For estimating and equalizing the optical channel, the pilots should be inserted into the OFDM signals before the OFDM waveforms are transmitted. Minimal pilot density and pilot position are important for the OFDM system for optimal performance. In [29], the pilot spacing used in each OFDM symbol was investigated and evaluated.

The maximum pilot spacing value of the OFDM symbol is Δp, as shown in Equation (4) [20].

(4) $$\Delta p\leq \frac{N\Delta f}{2\tau /T_{s}}$$

where *N* stands for the OFDM symbol, Δf is the frequency spacing between the subcarriers, NΔf is the OFDM bandwidth, τ is the time delay, and $T_{s}$ is the spatial sampling period.

**Figure 4:**
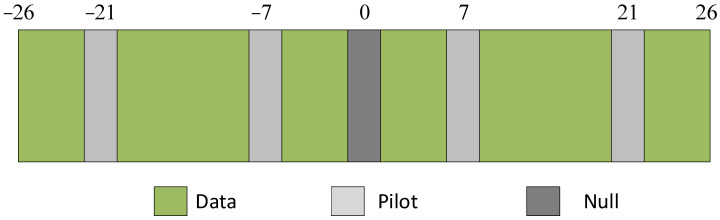
Example of a pilot position in the rolling-shutter OFDM symbol.

The pilot spacing needs to be short for suitable interpolation performance. However, the estimation performance is not relative to the number of pilots. If the pilots are too close together, the system performance is reduced because they do not carry the desirable information. Figure 4 shows an example of the pilot positions.

- *Channel Equalization*

Channel equalization is a procedure for reducing amplitude and phase alterations. During channel equalization, the channel effect is decreased to increase system performance. The equalization technique is then applied to balance the tradeoff between the efficiency and complexity of the processes [30]. For example, this model has two adjacent pilot points: $H_{0},H_{1}$. Based on linear interpolation, the H(x) point between $H_{0}$ and $H_{1}$ is depicted as in Equation (5):

(5) $$H(x)=H_{0}+(x-x_{0}).\frac{H_{1}-H_{0}}{x_{1}-x_{0}}\;\mathrm{with}\;0\leq x\leq 1$$

(6) $$Y_{equalized}=\frac{Y_{non\_equalized}}{H}$$

- *Rolling-Shutter OFDM Packet*

**Figure 5:**
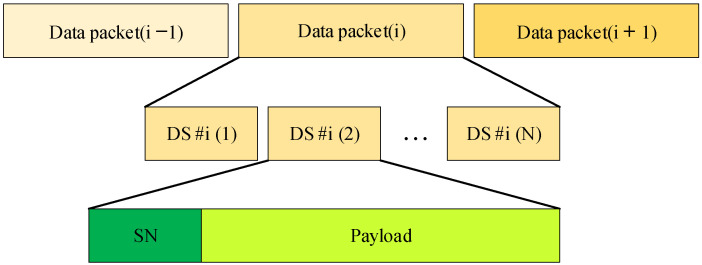
Data frame structure of the rolling-shutter OFDM scheme.

The frame-rate variation is an important parameter in the OCC system. In most cases, it is supposed that a camera’s frame rate is fixed (e.g., 30 fps or 1000 fps). In fact, all cameras have their own frame rates that differ based on the technical parameters. These parameters are unpredictable, making it even more difficult to synchronize the transmitter and receiver. To resolve this issue, a sequence number part is inserted into each OFDM packet, as shown in Figure 5. This assists the receiver side with assembling the packets in order and detecting any missing packets. The serial number of a packet is represented by SN. We can categorize situations based on the transmitter’s packet rate and camera’s frame rate into two scenarios. Case 1 pertains to undersampling, in which the camera’s frame rate is lower than the transmitter’s (LED) packet rate. Case 2 corresponds to oversampling, in which the camera’s frame rate significantly exceeds the packet rate of the transmitter. Our suggested data frame arrangement comprises numerous data packet frames, with each data subpacket (DS) containing payload data and a sequence number (SN). The DS components consist of multiple units. The SN serves as sequence information for a data packet, aiding a receiver in determining the arrival status of a new payload in situations with variable oversampling and detecting any missed payloads during undersampling conditions.

- *M-FSK Packet*

**Figure 6:**
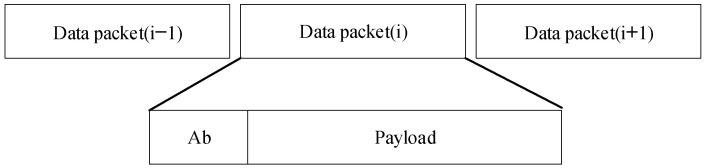
Data frame structure of the M-FSK scheme.

The M-FSK modulation data structure is as shown in Figure 6 and includes two parts: the asynchronous bit (representing the clock information of the data packet) for synchronization and payload for communication data. This was proposed by us in [5] as the CM-FSK scheme. The Ab bit is generated by a specific frequency of the optical light source. The data packet length should consider the image sensor frame rate and application scenario. The preamble frequency is generated by a specific frequency of the optical light source to synchronize the head and tail of the data packet, as represented by $f_{0}$ and $f_{5}$ in Table 1.

- *OFDM Symbol Synchronization*

Before performing further activities required by the OFDM system, such as frequency synchronization and channel estimation, the symbol’s correct starting point must be determined. The goal of symbol synchronization is to achieve the start point of the OFDM symbol. In this study, we use two waveforms to transmit data based on a single LED to reduce the confusion between two waveforms. The M-FSK waveform received by a smartphone can be easily detected and decoded by counting the row pixels between the “ON” and “OFF” statuses of M-FSK, as proposed in [5]. However, in the high-speed stream, we receive hybrid (O-OFDM and M-FSK) waveforms. To decode the OFDM signal in the hybrid waveform, we split the OFDM symbol from the hybrid waveform; then, the start of OFDM symbol detection is important before decoding the data. Van de Beek was proposed to estimate of time and frequency offset of OFDM technology in 1997 [31]. In [32], the authors proposed a non-data-aided algorithm based on ML to estimate timing and carrier frequency offset. In this work, to obtain good performance, we introduced the Van de Beek method for the real-time detection of the O-OFDM symbol’s frame start, which relies on correlation with the CP part.

- *Hybrid Waveforms*

**Figure 7:**
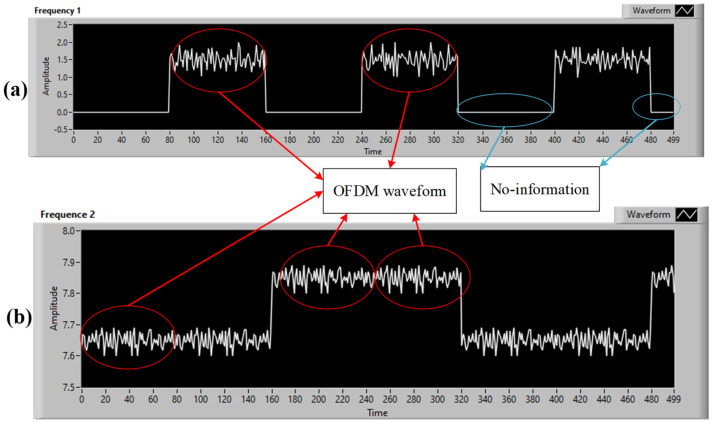
Hybrid waveform: (**a**) OFDM symbols embedded only in the high period of the FSK signal and (**b**) OFDM symbols embedded in the high and low periods of the FSK signal.

As mentioned in [20], the RS-OFDM scheme was designed based on intensity modulation/direct detection. It is applied to generate a multicarrier waveform, which is then converted to a voltage signage for driving the LED light source. With M-FSK, the data are transmitted based on different frequencies of the on/off statuses of the LED lamp. On the receiver side, based on the values and ranges of the on/off strips in the images, it is simple to decode the data. There are two options available to combine the FSK and OFDM waveforms. Case 1: As shown in Figure 7a, only the OFDM symbols will be placed in the high period of the FSK signal as the FSK signal is based on the LED’s on and off statuses. Because the value is 0 V in the FSK signal’s low period, no OFDM signals can be placed there. Case 2: The conventional M-FSK scheme will be updated. Two intensities are used to describe the on/off statuses on the transmitter side. For example, 5 V and 10 V are used to describe the on/off statuses instead of 0 V and 10 V in the conventional M-FSK scheme. Therefore, the OFDM symbols can be placed in both the high and low FSK signal periods. In case 2, the rate of the high data stream is increased by two times as compared to that of case 1. The hybrid waveform is shown in Figure 7b.

As mentioned previously, the hybrid waveform is created using two waveforms: M-FSK and RS-OFDM. Therefore, the M-FSK frequencies must carefully consider the clock rate of the RS-OFDM and OFDM symbol length (shown in Table 1). Table 1 illustrates the relationship between the clock rate and FSK frequencies. Because n OFDM symbols (n = 1, 2, 3, 4, …) are required in each period of the FSK signal, the $f_{0}$ illustrate the preamble frequency of the M-FSK signal. This signal is calculated based on the clock rate and length of the OFDM symbol as follows:

(7) $$f_{0}=\frac{f_{clock-rate}}{N_{OFDM\_frame}}$$

(8) $$f_{n}=\frac{f_{0}}{n+1}$$

The clock rate of the hybrid scheme is $f_{clock-rate}$, length of the OFDM frame is $N_{OFDM\_frame}$, and n is the number of OFDM symbols in each period (low or high) of the FSK signal (n = 1, 2, 3, …). The cycle of a hybrid signal has two cases (mentioned above). These are shown in Equations (9) and (10).

(9) $$s_{n}(t_{k})=\left\{\begin{array}{l} x_{OFDM,l}(t)+\ldots +x_{OFDM,l+n-1}(t+(n-1).\frac{T}{2.n})+A_{1}\\ 0 \end{array}\right.\;\mathrm{with}\;\begin{array}{l} 0\leq t_{k}<T/2\\ T/2\leq t_{k}<T \end{array}$$

(10) $$s_{n}(t_{k})=\left\{\begin{array}{l} x_{OFDM,l}(t)+\ldots +x_{OFDM,l+n-1}(t+(n-1).\frac{T}{2.n})+A_{1}\\ x_{OFDM,l+n}(t+n.\frac{T}{2.n})+\ldots +x_{OFDM,l+2n-1}(t+(2n-1)\frac{T}{2.n})+A_{2} \end{array}\right.\;\mathrm{with}\;\begin{array}{l} 0\leq t_{k}<T/2\\ T/2\leq t_{k}<T \end{array}$$

Here, $x_{OFDM,l}(t)$ is the $l^{th}$ OFDM symbol, and *n* is the number of OFDM symbols in each FSK signal period; *T* is the cycle of FSK waveforms. The two direct current voltage bias values are $A_{1},A_{2}$ ($A_{1}>A_{2}$) for the OFDM symbols. As is shown in Table 1, $s_{n}(t_{k})$ is the $f_{n-1}$ waveform. As noted in Table 1, the six frequencies correspond to n = 1, 2, …,6. The cycle of the OFDM symbol is $T_{OFDM}$. The relationship between the cycle of OFDM symbol and cycle of M-FSK waveform, *T*, is as in Equation (11).

(11) $$T_{OFDM}=T.\frac{1}{2n}$$

To guarantee a flicker-free condition, the full hybrid waveform (multiple waveform cycles) is expressed as follows:

(12) $$s_{n}(t)=\sum_{k=0}^{M}s_{n}(t_{k}+kT)$$

As shown in Equations (9) and (10), it is assumed that the OFDM symbol’s cycle is constant, so the cycle of the FSK signal is based on the number of OFDM symbols in each high period of the FSK signal. Accordingly, the frequencies of the FSK signal are shown in Table 1. The relationships between the optical clock rate (carrier frequency) and FSK scheme frequencies are depicted in Table 1.

## 3. Implementation Results

**Figure 8:**
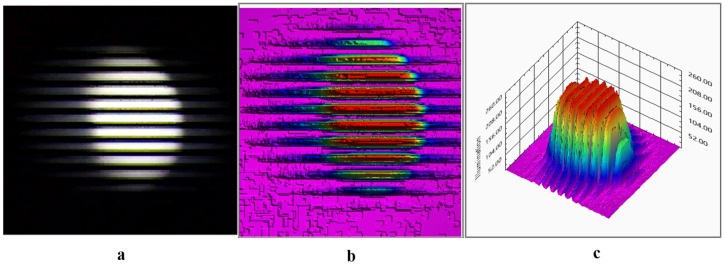
Performance analysis of the proposed hybrid scheme with a single LED: (**a**) original captured images frame; (**b**) top view of the illumination profile; and (**c**) quantized intensity profile at a distance of 50 cm using a smartphone camera.

**Figure 9:**
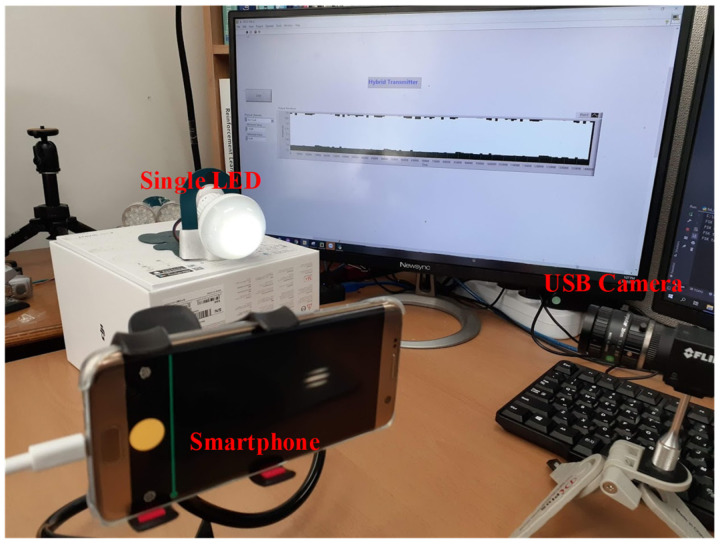
Setup of the hybrid OCC system.

The hybrid OCC scheme proposed in this study was constructed several times with different cameras to verify the frame variation effects. Figure 8 presents the original captured image frame, top view of the illumination profile, and quantized intensity profile with the proposed hybrid OCC waveform at distance of 50 cm. Based on Figure 8, the M-FSK signal can be easy to decode using a zero-crossing algorithm with a hybrid scheme because the space between the ON/OFF strips is long enough. The experimental setup of the proposed scheme is depicted in Figure 9. The outputs of the hybrid system were created and provided by the NI USB-6351 DAQ board. An LED lamp (10 V, 5 W) was used to display the hybrid waveform. The LED was connected to the NI USB-6351 DAQ board. In a receiver side, we have two cameras (smartphone and USB camera) at a distance of 50 cm; one is a USB camera (FL3-U3-132C-CS, frame rate 60 fps, focal lens 15 mm, image resolution 1440 × 1080), and the other is a smartphone camera (Samsung Galaxy S7 Edge, frame rate 240 fps, image resolution 1028 × 960).

**Figure 10:**
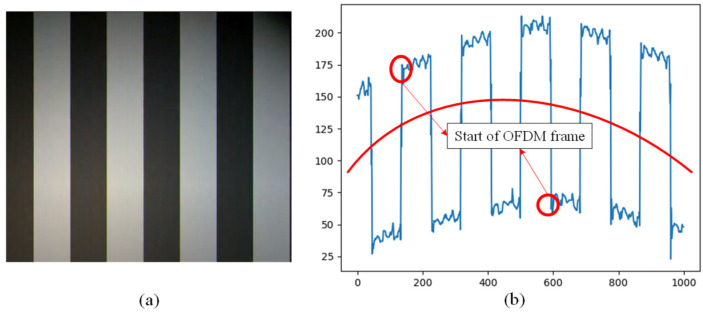
Received signals by the smartphone camera at 1 m: (**a**) original pattern and (**b**) hybrid waveform.

**Figure 11:**
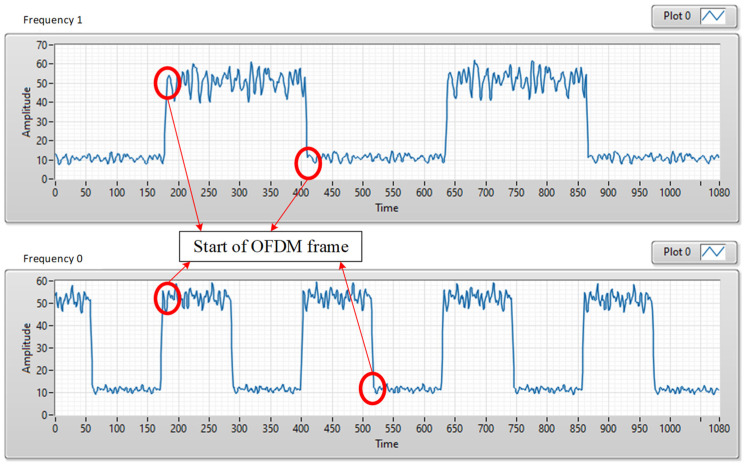
Received signals by the USB camera FL3-U3-132C-CS (PointGrey rolling-shutter camera).

The signals received by the smartphone camera are shown in Figure 10, and the signals received by the USB camera are shown in Figure 11. Figure 10 shows the M-FSK signal, which is collected by smartphone camera at a distance of 1 m. Figure 10a shows the original pattern images, and Figure 10b displays the received waveform. Figure 11 demonstrates that the high-frequency OFDM signals are carried by both the high and low levels of the FSK signals. The starting point of the OFDM symbol can be detected accurately in real time using the Van de Beek algorithm, and the OFDM signal can be removed from the hybrid waveform before decoding. Then, the performances of the hybrid system in the two data streams can be guaranteed. The smartphone camera easily decodes the FSK signal. As shown in Figure 10 and Figure 11, the M-FSK receiver can easily decode data by measuring the black and white strips in the received images, but we have to split the OFDM symbol within the hybrid waveform in the OFDM receiver. Based on the correction between the CP parts in each of the OFDM symbols, we can find the start point of the OFDM symbol in real time to split and decode the OFDM waveform easily from the received hybrid waveform. As shown in Figure 11, we can easily separate the OFDM waveform from the received hybrid waveform after detecting the start of frame using the Van de Beek algorithm. As shown above, when the OFDM waveform is put in the ON/OFF level of the M-FSK signal, we can achieve higher data rate.

In the low data stream, a 4-FSK modulation scheme was applied. The detailed specifications of the 4-FSK scheme are presented with its six frequencies in Table 1. RS-OFDM scheme was applied (relationship between OFDM modulation and 4-FSK scheme is shown in Table 1). The length of SN was selected to be compatible with the asynchronous processes, such as asynchronous decoding, detection of missing data segments, and data merging technique. Table 2 presents the various experimental results and parameters. The hybrid OCC system experiments involved assessing the camera’s frame-rate variations, which ranged between 50 fps and 60 fps. With the low data rate stream, we can achieve a data rate of up to 80 bps, which is suitable for localization application. In the high data rate stream, we can achieve a speed of 5.120 kbps using the DCO-OFDM scheme.

**Figure 12:**
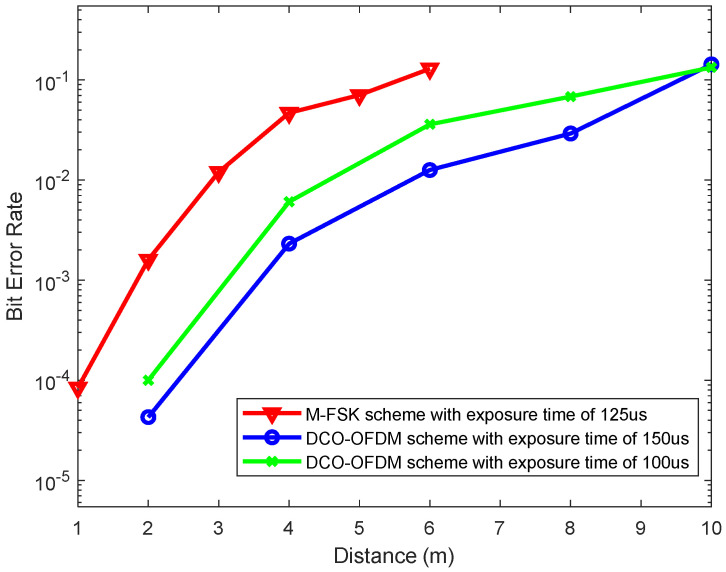
Bit error rates of the hybrid OCC system for different communication distances.

Figure 12 shows the hybrid OCC system’s performance results for different communication distances and exposure times in the indoor scenario. With M-FSK using a Samsung Galaxy S7 camera, we set up a demonstration with different distances ranging from 1 m to 6 m and measured the BER values to analyze the OCC system performance. The bit error rate values were obtained up to $10^{-4}$ due to limitations caused by the camera parameters (focal length, exposure time, etc.) of the smartphone. With the DCO-OFDM scheme, we deployed a USB smart camera as the receiver with a good focal length and exposure time, which allowed us to achieve a greater distance (from 2 m to 10 m). The bit error rate values were obtained up to $10^{-5}$. Figure 12 demonstrates that the M-FSK scheme achieved a BER of $10^{-4}$ at a communication distance of 1 m using the Samsung S7 Edge camera. The DCO-OFDM scheme achieved a BER of $10^{-3}$ at a communication distance of 4 m with the PointGrey rolling-shutter camera with a focal length of 16 mm. As mentioned previously, the pixel SNR values are regulated by controlling the exposure times to increase the communication distances. However, the shutter speeds can cause fuzziness on the receiver side, reducing the communication bandwidth. Using the C-mount lens specification for a flange back distance of 16 mm for the FL3-U3-132C-CS camera, we can achieve a communication distance of 10 m. As mentioned previously, we can increase the exposure time to increase the communication distance, but the communication bandwidth is reduced, thereby reducing the total noise power spread over the bandwidth. Hence, we utilized the forward error correction technique to increase the hybrid OCC performance and mitigate the larger fraction of fuzzy states. The proposed hybrid scheme was implemented with a single LED at a communication distance of 0.5 m (our demonstration video (Supplementary Material) can be found online at https://youtu.be/xaEDnNNrjW0 (accessed on 20 November 2023)).

## 4. Conclusions

This work presents and discusses a hybrid scheme using OCC that integrates two waveforms transmitted from a single transmitter LED and received by two different rolling camera devices. A smart camera with a high-resolution image sensor captures the high-frequency signal, and a low-resolution image sensor smartphone camera captures the low-frequency signal. This scheme enables various applications (two waveforms with two data streams) with low cost and low complexity owing to the use of a single LED to transmit two different data streams for different services. In the proposed scheme, M-FSK modulation was utilized for the low-rate OCC stream, whereas the RS-OFDM scheme was utilized for the high-rate OCC stream. The RS-OFDM symbols increase the hybrid system’s data rate using both the high and low periods of the FSK waveform. The relationship between the FSK and OFDM signals was also discussed herein to highlight this study’s contributions. Additionally, a frame structure was proposed using sequence numbers and Ab bits in the OFDM and FSK signals to mitigate the frame-rate variation effect, which is a critical phenomenon in OCC systems.

## Figures and Tables

**Table 1:** 4-FSK encoding table.

A Packet of Bits Input	Frequency Output
Preamble 1	$f_{0}$
00	$f_{1}=\frac{f_{0}}{2}$
01	$f_{2}=\frac{f_{0}}{3}$
10	$f_{3}=\frac{f_{0}}{4}$
11	$f_{4}=\frac{f_{0}}{5}$
Preamble 2 (Ab bit frequency)	$f_{5}=\frac{f_{0}}{6}$

**Table 2:** Hybrid OCC system parameters.

Tx Side		
Optical clock rate	19.448 kHz	43.413 kHz
OFDM symbol length	64	128
FEC	RS (15, 11)	
Packet rate	20 packet/s	
LED type	12 V, 2.5 W	
**Rx Side**		
Camera type	PointGrey rolling-shutter camera	
Camera frame rate	60 fps	
Throughput	2.560 kbps	5.120 kbps
Camera type	Smartphone camera (Samsung S7 Edge)	
Camera frame rate	240 fps	
Throughput	40 bps	80 bps

## Data Availability

Data are contained within the article and Supplementary Material.

## Supplementary Materials

The following are available online at https://www.mdpi.com/article/10.3390/s24010300/s1. The supplementary material video shows the implementation of our proposal scheme at the distance of 0.5 m.

## References

[1] Tan K.-S., Hinberg I., Wadhwani J. Electromagnetic interference in medical devices: Health Canada’s past and current perspectives and activities. Proceedings of the 2001 IEEE EMC International Symposium, Symposium Record, International Symposium on Electromagnetic Compatibility (Cat. No.01CH37161).

[2] Roberts-Intel R.D., Kyu S. (2012). IEEE 802.15.7 visible light communication: Modulation and dimming support. IEEE Commun. Mag..

[3] An Overview on High-Speed Optical Wireless/Light Communications. https://mentor.ieee.org/802.11/dcn/17/11-17-0962-02-00lc-an-overview-on-high-speed-optical-wireless-light-communications.pdf.

[4] (2011). IEEE Standard for Local and Metropolitan Area Networks—Part 15.7: Short-Range Wireless Optical Communication Using Visible Light.

[5] (2018). IEEE Standard for Local and Metropolitan Area Networks—Part 15.7: Short-Range Wireless Optical Communication.

[6] Cahyadi W.A., Chung Y.H., Ghassemlooy Z., Hassan N.B. (2020). Optical camera communications: Principles, modulations, potential and challenges. Electronics.

[7] Pham T.L., Nguyen T., Thieu M.D., Nguyen H., Nguyen H., Jang Y.M. An Artificial Intelligence-based Error Correction for Optical Camera Communication. Proceedings of the 2019 Eleventh International Conference on Ubiquitous and Future Networks (ICUFN).

[8] Huang R., Yamazato T. (2023). A Review on Image Sensor Communication and Its Applications to Vehicles. Photonics.

[9] Grand View Research (2023). Smart Transportation Market Size, Share & Trends Analysis Report By Solution (Ticketing Management System, Parking Management System, Integrated Supervision System), by Service, by Region, and Segment Forecasts, 2023–2030. https://www.grandviewresearch.com/industry-analysis/smart-transportation-market.

[10] Celik A., Romdhane I., Kaddoum G., Eltawil A.M. (2022). A Top-Down Survey on Optical Wireless Communications for the Internet of Things. IEEE Commun. Surv. Tutor..

[11] Aguiar-Castillo L., Guerra V., Rufo J., Rabadan J., Perez-Jimenez R. (2021). Survey on Optical Wireless Communications-Based Services Applied to the Tourism Industry: Potentials and Challenges. Sensors.

[12] Chang Y.H., Tsai S.Y., Chow C.W., Wang C.C., Tsai D.C., Liu Y., Yeh C.H. (2023). Unmanned-aerial-vehicle based optical camera communication system using light-diffusing fiber and rolling-shutter image-sensor. Opt. Express.

[13] Teli S., Cahyadi W.A., Chung Y.H. (2018). High-speed optical camera V2V communications using selective capture. Photonic Netw. Commun..

[14] Feng M., Wang Y., Li M., Liu S., Huang G., Li P. (2023). Design of OCC Indoor Positioning System Based on Flat Panel Light and Angle Sensor Assistance. Appl. Sci..

[15] White I., Curry E., Borah D.K., Stochaj S.J., Tang W. (2019). An optical spatial localization algorithm using single temporal difference image sensor. IEEE Sens. Lett..

[16] Van Hoa N., Nguyen H., Nguyen C.H., Jang Y.M. OCC Technology-based Developing IoT Network. Proceedings of the 2020 International Conference on Information and Communication Technology Convergence (ICTC).

[17] Teli S.R., Guerra-Yanez C., Icaza V.M., Perez-Jimenez R., Ghassemlooy Z., Zvanovec S. (2023). Hybrid Optical Wireless Communication for Versatile IoT Applications: Data Rate Improvement and Analysis. IEEE Access.

[18] Teli S.R., Chvojka P., Vítek S., Zvanovec S., Perez-Jimenez R., Ghassemlooy Z. (2022). A SIMO hybrid visible-light communication system for optical IoT. IEEE Internet Things J..

[19] Nguyen T., Thieu M.D., Jang Y.M. (2019). 2D-OFDM for optical camera communication: Principle and implementation. IEEE Access.

[20] Nguyen H., Nguyen V.L., Tran D.H., Jang Y.M. (2022). Rolling Shutter OFDM Scheme for Optical Camera Communication Consid-ering Mobility Environment Based on Deep Learning. Appl. Sci..

[21] Nguyen D.T., Park S., Chae Y., Park Y. (2019). VLC/OCC hybrid optical wireless systems for versatile indoor applications. IEEE Access.

[22] Yang F., Gao J., Liu S. (2016). Novel visible light communication approach based on hybrid OOK and ACO-OFDM. IEEE Photonics Technol. Lett..

[23] Xu C., Xiang L., An J., Dong C., Sugiura S., Maunder R.G., Yang L.L., Hanzo L. (2023). OTFS-Aided RIS-Assisted SAGIN Systems Outperform Their OFDM Counterparts in Doubly Selective High-Doppler Scenarios. IEEE Internet Things J..

[24] Abdulwali J., Boussakta S. (2022). Visible Light Communication: An Investigation of LED Non-Linearity Effects on VLC Utilising C-OFDM. Photonics.

[25] Armstrong J., Schmidt B.J.C. (2008). Comparison of Asymmetrically Clipped Optical OFDM and DC-Biased Optical OFDM in AWGN. IEEE Commun. Lett..

[26] Hosseini H., Fisal N., Syed-Yusof S.K. (2010). Wavelet packet-based multicarrier modulation for cognitive UWB systems. Signal Process. Int. J..

[27] Huang W., Gong C., Xu Z. (2015). System and waveform design for wavelet packet division multiplexing-based visible light communications. J. Light. Technol..

[28] Rahman M.H., Sejan M.A.S., Chung W.Y. Long-Distance Real-Time Rolling Shutter Optical Camera Communication Using MFSK Modulation Technique. Proceedings of the International Conference on Intelligent Human Computer Interaction.

[29] Hanzo L., Munster M., Choi B.J., Keller T. (2003). OFDM and MC-CDMA for Broadband Multi-User Communications WLANs and Broadcasting.

[30] Dong X., Lu W., Soong A.C.K. (2007). Linear interpolation in pilot symbol assisted channel estimation for OFDM. IEEE Trans. Wirel. Commun..

[31] Van de Beek J., Sandell M., Borjesson P.O. (1997). ML estimation of time and frequency offset in OFDM systems. IEEE Trans. Signal Process..

[32] An J., Gan L., Liao H. A non-data-aided algorithm based on ML for OFDM synchronization. Proceedings of the 2018 International Conference on Electronics Technology (ICET).

